# Cognitive–Mathematics Relations: A Meta-Analysis of Norm-Referenced Standardized Test Batteries

**DOI:** 10.3390/jintelligence14070142

**Published:** 2026-07-06

**Authors:** Christopher R. Niileksela, Daniel B. Hajovsky, Francisco Philippe Cocar-Montenegro, William Joel Schneider, Dawn P. Flanagan, Vincent C. Alfonso

**Affiliations:** 1 Department of Educational Psychology, University of Kansas, Lawrence, KS 66045, USA; francisco.cocar@ku.edu; 2 Department of Educational Psychology, Texas A&M University, College Station, TX 77845, USA; dhajovsky@tamu.edu; 3 Psychological Studies in Education, Temple University, Philadelphia, PA 19122, USA; schneider@temple.edu; 4 Department of Psychology, St. John’s University, New York City, NY 11439, USA; flanagad@stjohns.edu; 5 College for Education and Engaged Learning, Montclair State University, Montclair, NJ 07043, USA; alfonsov@montclair.edu

**Keywords:** cognitive abilities, mathematical skills, cognitive–achievement relations, CHC theory, meta-structural equation modeling

## Abstract

The relationships between cognitive abilities and mathematics skills are important to examine to help clarify how cognitive abilities promote mathematics development and inform why some individuals have difficulty acquiring mathematics skills. Meta-analyses of cognitive–mathematics relations can help summarize this research across a wide variety of test batteries and samples. This study compiled data from technical manuals of 122 norm-referenced standardized test batteries and included over 47,000 correlations from over 550 correlation matrices to summarize cognitive–mathematics relations using meta-analysis. The meta-analytic correlations were used to estimate cognitive–mathematics relations in an integrated model of cognitive abilities and mathematics skills. Fluid reasoning and comprehension-knowledge were two of the most consistent cognitive predictors of mathematics skills, and foundational mathematics skills (e.g., number sense and math fluency) were consistent predictors of advanced mathematics skills (e.g., math problem solving). A supplemental analysis examined how several narrow cognitive abilities (e.g., lexical knowledge, induction) predict mathematics skills. Results suggested that some narrow cognitive abilities, like general knowledge and general sequential reasoning, were consistent predictors of mathematics skills. This study summarizes a large amount of data from norm-referenced standardized test batteries to clarify how cognitive abilities predict mathematics skills. These results can inform both theoretical models of mathematics development and practical strategies when evaluating individuals who may have difficulty acquiring mathematics skills.

## 1. Introduction

Developing mathematics skills is an essential component of professional and personal success. Mathematics skills are related to multiple life outcomes, including career and post-secondary success, leadership skills, and future income (Lubinski et al., 2014). While the importance of mathematics proficiency is well-established, educators face the significant challenge of improving outcomes in Science, Engineering, Technology, and Mathematics (STEM) fields to prepare children to become competent problem solvers who can apply their learning to global issues (Green et al., 2017), making it one of the most important skills for social progress and prosperity.

Mathematics may be challenging for students during school, and some students may require mathematics remediation or intervention. Consequently, it is helpful to identify cognitive abilities linked to mathematics skills so educators can better understand and develop learning contexts that target both mathematics skills and cognitive processes that may enhance mathematics achievement (Green et al., 2017). Cognitive abilities are one of the strongest predictors of mathematics skills (Berkowitz & Stern, 2018; Geary, 2011; Xenidou-Dervou et al., 2017). By understanding how cognitive abilities and mathematics are related, we can better identify the cognitive processes that underlie mathematics skill acquisition and development. With this knowledge, we may better understand why some students excel in mathematics while others have more difficulty with mathematics during school.

Researchers have examined the relations between cognitive abilities and mathematics using individual test batteries (e.g., Niileksela et al., 2025; Villeneuve et al., 2019), while others have used cross-battery approaches (e.g., Caemmerer et al., 2023). Several meta-analyses have examined the relations between cognitive abilities and mathematics, investigating a range of broad cognitive abilities (e.g., fluid reasoning, working memory, Amland et al., 2025; McGrew & Wendling, 2010; Zaboski et al., 2018). However, no meta-analyses have compiled data from available norm-referenced standardized test batteries to examine cognitive–mathematics relations. Norm-referenced standardized test batteries of cognitive abilities and mathematics skills that are used in practice have technical manuals that explain how the tests were developed, describe the reliability and validity evidence for those tests, and often include data from large samples of people that are representative of a specific population at a specific time (e.g., the population of the United States based on census data close to the time that the test was developed). This approach allows for the consolidation of cognitive–mathematics relations from a large number of tests that have been carefully designed, with carefully selected normative samples, and are commonly used in clinical and school-based practice. The purpose of this study is to present a comprehensive meta-analysis of cognitive–mathematics relations using correlation matrices from published, norm-referenced standardized test batteries of cognitive abilities and academic skills.

### 1.1. Theoretical Anchors

This study is grounded in theories of mathematical cognition and the structure of cognitive abilities. Specifically, mathematical cognition is conceptually grounded in the pathways model (Dehaene, 1997, 2011; Dehaene et al., 2005; LeFevre et al., 2010) and the hierarchy of mathematical competencies (Xu et al., 2023). The structure of cognitive abilities is grounded in the Cattell-Horn-Carroll Theory of Cognitive Abilities (Schneider & McGrew, 2018). The cognitive predictors of mathematics have received some focus in the literature, but not as much as reading (e.g., Hajovsky et al., 2025). The development of reading skills is well understood, and there is a strong research base that informs how reading develops in a variety of languages (Ziegler & Goswami, 2005). Although reading research focuses on a few core linguistic processes (Gough & Tunmer, 1986), mathematics is often viewed as a multidimensional construct, involving a wider variety of cognitive abilities that change as mathematical tasks become more complex.

### 1.2. Development of Mathematics Cognition

Like the simple view of reading (Gough & Tunmer, 1986) for understanding reading comprehension, there are models that help describe and explain how mathematics skills develop across childhood and adolescence. The two models underpinning mathematics cognition in this study are: (1) the pathways model by Dehaene et al. (2005), which describes neurocognitive mechanisms related to mathematics, and (2) the hierarchy of mathematical competencies (Xu et al., 2023), which describes how mathematics skills are hierarchically ordered, where basic mathematics skills serve as the foundation to more advanced mathematics skills.

The pathways model (Dehaene et al., 2005) includes three pathways and proposes neural pathways in the parietal lobe working independently of each other to contribute toward acquiring mathematical knowledge. First, the linguistic pathway refers to the retrieval of mathematical operations or equations and language specific to mathematical knowledge. Second, the quantitative pathway involves quantitative tasks through a non-linguistic approximation of number magnitude, or the approximate number system (Geary, 2011). Third, the spatial attention pathway is congruent with nonverbal working memory and involves recognizing Arabic digits, but is not specifically connected to specific numerical tasks, but connecting written numerals to magnitudes (Dehaene et al., 2005). LeFevre et al. (2010) suggested that the linguistic pathway was primarily related to the symbolic number system, which was then related to a variety of mathematics skills (e.g., calculation, geometry, magnitude comparison). The quantitative pathway was primarily related to number magnitude and subsequently related to other math skills (e.g., calculations, magnitude comparisons), while the spatial attention pathway had broader relationships across most areas of early numeracy and mathematics skills.

While the pathways model focuses on cognitive processes that are related to mathematics, the hierarchy of mathematical competencies describes how mathematics skills are ordinally related to each other. This distinction is important because it suggests that foundational mathematical skills are essential predictors of advanced and complex mathematical skills (e.g., Villeneuve et al., 2019). In this model, fundamental numeracy refers to the ability to decode symbolic knowledge through cardinal and ordinal relations. (Xu et al., 2023). These fundamental skills serve as a foundation for more complex competencies. Next, additive and multiplicative relations involve the ability to solve addition or multiplication problems using whole numbers and build on an individual’s fundamental numeracy capacity. Additive relations include addition and subtraction, which complement each other (e.g., 9 + 2 = 11, 11 − 9 = 2), and multiplicative relations, which include multiplication and division, also complement each other (e.g., 4 × 3 = 12, 12/4 = 3) (Xu et al., 2023). Additive relations are embedded within multiplicative relations because a person must understand the concept of addition prior to understanding the concept of multiplication. Knowledge of these two processes enhances an individual’s ability to integrate all additive and multiplicative operations (Xu et al., 2023). Next, rational numbers refer to the ability to reason when numbers are represented as fractions and can be understood in terms of multiplication and division (e.g., knowing that ½ of 10 is five is the same as understanding that 10 is twice as large as 5) (Xu et al., 2023). Finally, all mathematics skills are embedded within algebraic knowledge, which is the ability to manipulate symbols in conjunction with fractions and multiplicative and additive operations. This is a disjunctive model, where insufficient ability in any previous level of the model may increase the difficulty in completing algebraic equations. (Xu et al., 2023).

### 1.3. Cattell-Horn-Carroll Theory on the Structure of Human Cognitive Abilities

The Cattell-Horn-Carroll Theory of Intelligence (CHC) is an empirically based taxonomy of human cognitive abilities (Schneider & McGrew, 2018). We use CHC theory as a foundational taxonomy for classifying the abilities measured by different cognitive and mathematics tests. CHC theory does not explain or describe why mathematics skills develop; it helps classify a heterogeneous set of tests into a well-established taxonomy of cognitive abilities and mathematics skills. In CHC theory, abilities are organized into three hierarchical levels. The most general level includes the “*g*” factor at the top, or stratum III (Schneider & McGrew, 2018). Second, there are 17 broad abilities in CHC theory, but for this study, the focus will be on nine of those abilities. Eight cognitive abilities referred to as “broad abilities” constitute stratum II, which represent different aspects of information processing including comprehension-knowledge (G*c*, a person’s ability to understand and use knowledge that is culturally significant along with concepts and skills learned through education, life experience, and culture), fluid reasoning (G*f*, the ability to solve novel problems without using previously learned information, habits, and/or schemas), short-term working memory (G*wm*, the ability to maintain and manipulate visual and/or auditory stimuli for a specific purpose), long-term storage (G*l*, the ability to store various types of information in long-term memory), retrieval fluency (G*r*, the ability to rapidly and accurately access information in long-term memory), visual processing (G*v*, the ability to use of mental representations to address problems, recognize differences, manipulate, and remember nonlinguistic mental images), auditory processing (G*a*, the ability to distinguish, retain, analyze, and work creatively on sounds, including language) and processing speed (G*s*, the ability to attend to and rapidly perform simple cognitive tasks with accuracy and fluency). Third, there are more than 70 specific abilities that underlie the broad abilities and are referred to as “narrow abilities” that constitute stratum I (Schneider & McGrew, 2018). These represent specific aspects of human cognitive processing. Navigating these cognitive abilities aids in understanding the nature of human intelligence and informing pertinent applications to psychological and educational practices. See Schneider and McGrew (2018) for comprehensive definitions of each broad and narrow ability. Grounding research in cognitive abilities strengthens the links between education and psychology as educators know more about how to adjust educational plans in response to individual differences in cognition (Warne, 2016).

CHC theory also includes reading/writing and mathematics as broad abilities within its taxonomy. There are several narrow mathematical abilities that are part of the CHC theory. Number Sense is conceptualized as an early capacity for representing and comparing quantities that provides a foundational scaffold for later symbolic mathematics (e.g., understanding the difference between three apples on a table and five apples on a table without knowledge of explicit verbal number systems). Neurocognitive and behavioral evidence support treating Number Sense as a distinct foundational skill that supports the development of formal mathematics, where preverbal approximate number representations and rapid magnitude comparisons appear early in development and predict later arithmetic skill (Dehaene, 1997, 2011; LeFevre et al., 2010). In this study, we map Number Sense onto the quantitative pathway and examine whether basic mathematics skills mediate relations between broad CHC abilities and higher-order mathematics outcomes, while acknowledging that these mappings reflect theoretical alignment rather than proof of temporal sequencing.

The narrow ability of math fluency is often combined with math calculation in test score composites (e.g., Woodcock–Johnson V, McGrew et al., 2025). Math fluency refers to the ability to apply mathematical procedures efficiently, effectively, and accurately, as well as transferring them to novel problems, typically around the accurate recall of math facts. Math calculation refers to the ability to solve mathematical operations and to solve calculation problems without any context (Floyd et al., 2003; Schrank & Wendling, 2018). Math knowledge refers primarily to general acquired knowledge regarding mathematics, which is more about the content knowledge aspect of mathematics rather than the procedural knowledge aspect of mathematics (Schneider & McGrew, 2018). Finally, Math Problem Solving refers to the application of reasoning and mathematical knowledge to solve real-world scenarios or meaningful problems (Schrank & Wendling, 2018). This may also include using existing knowledge of mathematical procedures and concepts in unique ways to solve practical problems. This is also referred to as procedural fluency in the mathematics education literature (NCTM, 2023).

### 1.4. Cognitive–Mathematics Relations in Previous Research

Several broad cognitive abilities aligned with CHC theory have consistently been related to mathematics skills. Comprehension-knowledge has consistently been found as an important predictor of mathematics (Benson et al., 2016; Caemmerer et al., 2018; Floyd et al., 2003; Niileksela et al., 2025; Villeneuve et al., 2019) and may help develop these skills by using learned concepts and vocabulary to support interpreting and analyzing mathematical problems. Comprehension-knowledge becomes more relevant to mathematical problem solving as students matriculate through school (Taub et al., 2008; Villeneuve et al., 2019). Fluid reasoning has also been consistently related to math achievement (e.g., Caemmerer et al., 2023; Green et al., 2017; Niileksela et al., 2025; Villeneuve et al., 2019). Fluid reasoning and math problem solving engage a shared cognitive process called relational reasoning, where reasoning skills are used to understand how different aspects of problems are related to one another. This aligns well with the narrow ability of quantitative reasoning (RQ) in CHC theory (Miller Singley & Bunge, 2018; Schneider & McGrew, 2018), which focuses on mathematical relations among stimuli rather than general relations among concepts or stimuli.

Short-term working memory has been related to mathematics skills in previous research (Bull et al., 2008; Jarvis & Gathercole, 2003; Van de Weijer-Bergsma et al., 2015; Villeneuve et al., 2019). Short-term working memory is moderately related to mathematical problem solving (Ji & Guo, 2023). Visual–spatial short-term memory (encoding and maintaining visual stimuli in short-term memory) and auditory short-term storage (encoding and maintaining verbal stimuli in short-term memory) have been related to mathematics (Schneider & McGrew, 2018) and predict math achievement because they help keep track of the steps while solving word problems and may help with the creation of mental representations of possible solutions. Short-term working memory becomes relatively less important for math problem solving as children matriculate through school (e.g., Villeneuve et al., 2019). Processing speed predicts mathematics skills (Geary, 2011; McGrew & Wendling, 2010; Taub et al., 2008), especially in younger children (Taub et al., 2008). Specifically, the narrow ability number facility (G*s*-N) is related to mathematical skill as it refers to the ability to rapidly and precisely work with numbers, spotting number patterns, and doing simple mathematical calculations (Schneider & McGrew, 2018).

Several cognitive abilities have been found to have small to moderate relations with mathematics, but the evidence has been less consistent. Visual processing appears to have a moderate relationship with mathematics (Atit et al., 2022), and it may be relevant to math computation across elementary and secondary school (Niileksela et al., 2025; Villeneuve et al., 2019). Long-term storage and retrieval fluency had been combined into a single long-term storage and retrieval factor in most previous research, and that had been identified as a predictor of math calculation and problem solving as students matriculate through school (Calderón-Tena & Caterino, 2016), but recent evidence has not suggested that either long-term storage or retrieval fluency is strongly related to mathematics. Finally, auditory processing has been related to mathematics, but not very consistently, and that relationship has been small when it is found (e.g., Amland et al., 2025). This relationship is likely due to the connection between phonological and language skills, since many tests of auditory processing focus on phonological skills over general auditory processing.

Although research has shown that some broad cognitive abilities are consistently related to mathematics, it is important to acknowledge that the relations between cognitive abilities and mathematics are sometimes moderated by contextual factors. For example, socioeconomic status (SES) has been identified as a contextual moderator. Evidence suggests there is a moderate positive relationship between SES and achievement (e.g., J. Liu et al., 2022; Sirin, 2005; White, 1982), where people with higher levels of SES tend to have higher levels of achievement. However, the relationship between fluid reasoning and mathematics may be moderated by SES, where there is a stronger relationship between fluid reasoning and mathematics for children from moderate to high levels of SES than for children from low levels of SES (Peng et al., 2019). Different types of environmental factors can have an influence on these relations that are more specific than SES. For instance, Blankson and Blair (2016) found that the relations between fluid reasoning and comprehension-knowledge were moderated by classroom quality, where kindergarten students who were in higher quality classrooms tended to show more improvement in mathematics skills if they had higher levels of fluid reasoning or comprehension-knowledge. Thus, it is important to interpret the results of this study and past studies when considering these contextual influences.

### 1.5. Meta-Analyses of Cognitive–Mathematics Relations

Several previously published systematic reviews and meta-analyses highlight the importance of synthesizing existing data to provide a comprehensive overview of predictors of mathematical ability (Amland et al., 2025; McGrew & Wendling, 2010; Zaboski et al., 2018). In a recent large meta-analysis by Amland et al. (2025), they synthesized cognitive factors underlying mathematical skills through a systematic review of the existing literature and a meta-analysis of the data extracted from 269 concurrent studies and 174 longitudinal studies. Their criterion encompassed concurrent and longitudinal studies where data were reported on at least 15 children and adolescents ages 4 to 16. The studies included in the meta-analysis reported a quantitative measure of mathematical skill and another measure that would serve as its predictor. From these studies, Amland et al. (2025) identified 2696 correlations of predictors of mathematical ability. Each predictor was classified under the pathways model of mathematical development (quantitative, linguistic, and spatial attention pathways) to create a comprehensive overview that would determine which factors predict mathematical achievement.

Amland et al. (2025) reported the meta-analytic correlations between cognitive factors and mathematics skills separately for concurrent and longitudinal samples. We focused on summarizing results from the cognitive–mathematics relations that are most relevant to the current study. From the concurrent samples, language skills, spatial ability, working memory, and nonverbal IQ were all moderately correlated with a variety of mathematics skills (e.g., most correlations between .25 and .50). The longitudinal samples also had moderate correlations between the same cognitive factors mentioned previously and a variety of mathematics skills, though most correlations were closer to .30.

In a second analysis, Amland et al. (2025) used the meta-analytic correlations to estimate a path model where different cognitive factors predicted arithmetic and word-problem-solving skills. In the concurrent samples, the approximate number system, digit knowledge, number word knowledge, phonological skills, and verbal working memory were statistically significant predictors of arithmetic problems, whereas digit knowledge, number word knowledge and nonverbal IQ were predictors of word problems. Similar results were found with the longitudinal samples, except that spatial ability predicted arithmetic problems and word problems, and language comprehension and visuo-spatial working memory predicted word problems.

From their results, non-symbolic number skills (referred to as Number Sense in the present study) had a smaller correlation with mathematical achievement compared to number skills. Number skills were the largest predictor of mathematical ability. Moreover, the authors also report that arithmetic skills were predicted by non-symbolic number skills and phonological skills; these correlations were small. Nonverbal IQ and language comprehension were strong predictors of word-problem ability. Lastly, only symbolic number skills predicted arithmetic and word problems.

### 1.6. Current Study

Previous research suggests several consistent patterns between cognitive abilities and mathematics skills, but a comprehensive meta-analysis of norm-referenced standardized test batteries has not been completed to synthesize this research and provide robust estimates of cognitive–mathematics relations. This study brings together hundreds of correlation matrices from norm-referenced standardized test batteries to estimate the meta-analytic correlations between cognitive abilities and mathematics skills and subsequently uses those correlations to estimate a model of cognitive–mathematics relations. Specifically, this study had two primary research questions:

1. What are the average correlations between CHC broad abilities (e.g., comprehension-knowledge, fluid reasoning) and narrow mathematics skills (e.g., calculation, math problem solving)?
2. Using the average correlations, what are the cognitive–mathematics relations in a meta-analytic structural equation model?

## 2. Methods

### 2.1. Correlation Matrices from Test Manuals

This meta-analysis is part of a broader effort to locate all norm-referenced standardized cognitive and academic test batteries that have been published (including tests that are currently and no longer in use), and compile correlation matrices included in the technical manuals of those tests. New and revised versions of test batteries that are published are added to this database as the technical manuals are made available. Test manuals for the current database of cognitive and academic test correlations were obtained from university-based clinics, and the manuals were reviewed for correlation matrices at the subtest level. Correlation matrices with data from normative and concurrent validity samples were extracted from norm-referenced standardized test battery manuals.

### 2.2. Constructs Included in the Meta-Analysis

For tests of cognitive ability, the analysis focused on broad CHC abilities, although tests were also classified by both broad and narrow CHC abilities (Schneider & McGrew, 2018). The eight broad abilities consistent with contemporary CHC theory that were included in this study were Comprehension-Knowledge (G*c*), Fluid Reasoning (G*f*), Visual Processing (G*v*), Auditory Processing (G*a*), Long-Term Storage (G*l*), Retrieval Fluency (G*r*), Short-Term Working Memory (G*wm*), and Processing Speed (G*s*). The narrow mathematics skills included Number Sense, Math Calculation Skills, Math Fluency, Math Knowledge, and Math Problem Solving. All correlations that included the eight broad CHC abilities and five narrow mathematics skills were the focus of this study.

### 2.3. Inclusion Criteria

Only correlations among subtests were used in this study. Inclusion criteria were (1) the correlation matrix had to include a correlation between two or more subtests (i.e., there were no correlations between composite scores or subtests and composite scores), and (2) the samples used to estimate the correlation matrices were from normative or concurrent validity samples. For normative samples, if a correlation matrix was included for the full sample and for age or grade-specific samples, the matrices for the age- or grade-specific samples were used over the correlation matrix. The inclusion of concurrent validity samples was to provide correlations across different test batteries, allowing for the inclusion of cross-battery correlations. If a concurrent validity correlation matrix used two versions of the same test (e.g., there were correlations between the Wechsler Adult Intelligence Scale—Third Edition and Wechsler Adult Intelligence Scale—Fourth Edition), then the correlations for the same subtests across those two batteries were not used (e.g., Symbol Search for both editions). These correlations were not included because the correlation between the same subtest on two versions of the same instrument would likely be inflated due to shared method variance. Finally, correlation matrices for clinical samples were not included in this study. While correlation matrices for these samples are not commonly reported in test manuals, if they were reported, they were not included in this study. The use of clinical samples may introduce systematic differences into the size of correlations due to restriction or expansion of range, so those correlation matrices were not used here.

### 2.4. Data Extraction and Coding of Subtests

Once the matrices were identified, several pieces of data were recorded. First, the name of the correlation matrix was specified (e.g., WISC-V Age 6 years 0 months to 6 years 11 months). Next, several pieces of information were extracted from the correlation matrix, including the name of the test battery or test batteries used for the correlation coefficient, the names of the two subtests included in the correlation, the correlation coefficient, and the sample size for each correlation pair. If a correlation matrix did not have the same sample size for each correlation pair, the sample size for each correlation in the matrix was recorded.

All tests from the correlation matrices were classified by broad and narrow CHC abilities. For tests of cognitive ability, the analysis focused on broad abilities, but tests were also classified by narrow abilities. All test classifications were based on the current CHC taxonomy, based on empirical work and expert consensus (e.g., Flanagan et al., 2025; LaForte et al., 2025, pp. 214–218; Schneider & McGrew, 2018). These classifications were determined using extensive examination of tests based on the CHC framework, informed by updated research. Test classifications are primarily based on the work of three authors (initials hidden for peer review). Tests were also classified as being either “good” or “poor” indicators of the broad and narrow CHC ability under which they were classified. Only subtests that were assessed to be “good” indicators of the broad CHC ability (for cognitive tests) and broad and narrow CHC abilities (for mathematics tests) were included in the meta-analysis. For example, the Woodcock–Johnson IV includes a test called Nonword Repetition, where the examinee hears a word made up of English-language phonemes, but it is not a real word. They must repeat the word to get the item right. This test measures both auditory processing and short-term working memory, and research indicates it loads on both factors (e.g., Niileksela et al., 2016). Thus, this test is a poor indicator of both auditory processing and short-term working memory because of the multiple cognitive processes required to complete the task. Other tests that primarily measure a single broad ability and have support from previous research as measuring one broad ability, such as Oral Vocabulary from the Woodcock–Johnson IV (a clear measure of Verbal Comprehension), were classified as “good” indicators of CHC abilities (Flanagan et al., 2013). The supplemental materials include a table of all test batteries used for the analysis, and a table of all the subtest names, correlation coefficients, sample sizes, and broad and narrow CHC ability categorizations for each correlation pair included in the analysis.

### 2.5. Stage 1: Estimating Meta-Analytic Correlations

A two-stage meta-analytic structural equation model (MASEM) was used in this study. In the first stage, the meta-analytic correlations among all cognitive and mathematics tests were estimated. Those correlations were compiled into a single correlation matrix that was used in the second stage, where a structural model was estimated to examine cognitive–mathematics relationships.

All meta-analytic correlations were estimated using the *metafor* package in R (Viechtbauer, 2010). Prior to analysis, all correlations were transformed using Fisher’s *Z*. Meta-analytic averages were then back-transformed to Pearson correlation coefficients. All meta-analytic correlations were estimated using a three-level random effects model. The first level is the within-study sampling error. When estimating the meta-analytic correlations, the within-study sampling error was used to weight the correlations, where studies with larger sample sizes (representing more precise correlation estimates) are more strongly weighted when estimating the meta-analytic average correlation. The inverse variance method was used for weighting (i.e., reciprocal of the squared standard error estimate, or (1/√(*N* − 3)^2^). The second level is the between-study variance. This random effect represents the distribution of correlations around the meta-analytic average, with the assumption that there is not one “true” correlation between two constructs, but there is a distribution of correlations around those two constructs. The third level accounts for within-study variance. This accounts for variance among tests that measure the same broad or narrow CHC ability that are clustered in the same correlation matrix. Many correlation matrices include multiple measures of the same CHC ability. For example, there are four Comprehension-Knowledge subtests on the WISC-V (i.e., Vocabulary, Similarities, Information, and Comprehension) and there are two Visual Spatial subtests on the WISC-V (i.e., Block Design and Visual Puzzles). This means there are eight different correlations between tests of Comprehension-Knowledge and Visual Processing in one correlation matrix. This third level of the random effects model estimates variance due to correlations measuring the same abilities in a single correlation matrix. All meta-analytic correlations were estimated using the restricted maximum likelihood estimator (RMLE), which works well for providing unbiased variance estimates for level 2 and level 3 of the random effects model and can be used with small and large sample sizes.

Finally, heterogeneity for the meta-analytic correlations was examined through the level 2 and 3 variance estimates (statistically significant variances indicating heterogeneity of correlations), as well as the *Q* and *I*^2^ statistics. Overall heterogeneity is estimated through the *Q* statistic, and *I*^2^ is an estimate of how much variance in correlations across different studies is due to true heterogeneity versus sampling error.

### 2.6. Stage 2: Using Meta-Analytic Correlations to Examine Cognitive–Mathematics Relations

The meta-analytic correlation matrix of cognitive abilities and mathematics skills was used to estimate a structural equation model (SEM) using M*plus* 7.4 (Muthén & Muthén, 1998–2015). An integrated model of mathematics skills was estimated for the second part of this study. This is consistent with the hierarchical model of mathematics competency, where foundational mathematics skills are embedded within complex mathematics skills. From a modeling perspective, this suggests that basic skills should be predictors of advanced mathematics skills, rather than only allowing mathematics skills to be correlated with each other. In this model, all mathematics skills were predicted by the eight broad CHC abilities. Then, mathematics skills were organized in a cascading fashion, where Number Sense was a predictor of Math Fluency, and then Number Sense and Math Fluency were predictors of Math Calculation. Those three mathematics skills predicted Math Knowledge, and all four of those mathematics skills predicted Math Problem Solving.

The sample size for the analysis was set by estimating the harmonic mean of sample sizes for each correlation pair and then estimating the average sample size of those harmonic means, which was *N* = 253 (Cheung, 2015; Viswesvaran & Ones, 1995). Ultimately, the primary focus was on the magnitude of the path coefficients over their statistical significance. Standardized path coefficients < .05 were interpreted as negligible, coefficients between .05 and .09 were interpreted as small, coefficients between .10 and .24 were interpreted as moderate, and coefficients ≥ .25 were interpreted as large (Keith, 2019).

## 3. Results

### 3.1. Descriptive Statistics

Overall, there were 47,231 correlations included in this meta-analysis. These correlations came from 552 different correlation matrices from 122 different test batteries, represented by 1060 subtests from the different test batteries. Table 1 shows the number of correlations included in the meta-analytic average correlation between each pair of variables, and Table 2 shows the number of correlation matrices used in the analyses for each pair of variables.

### 3.2. Meta-Analytic Correlations

**Figure 1:**
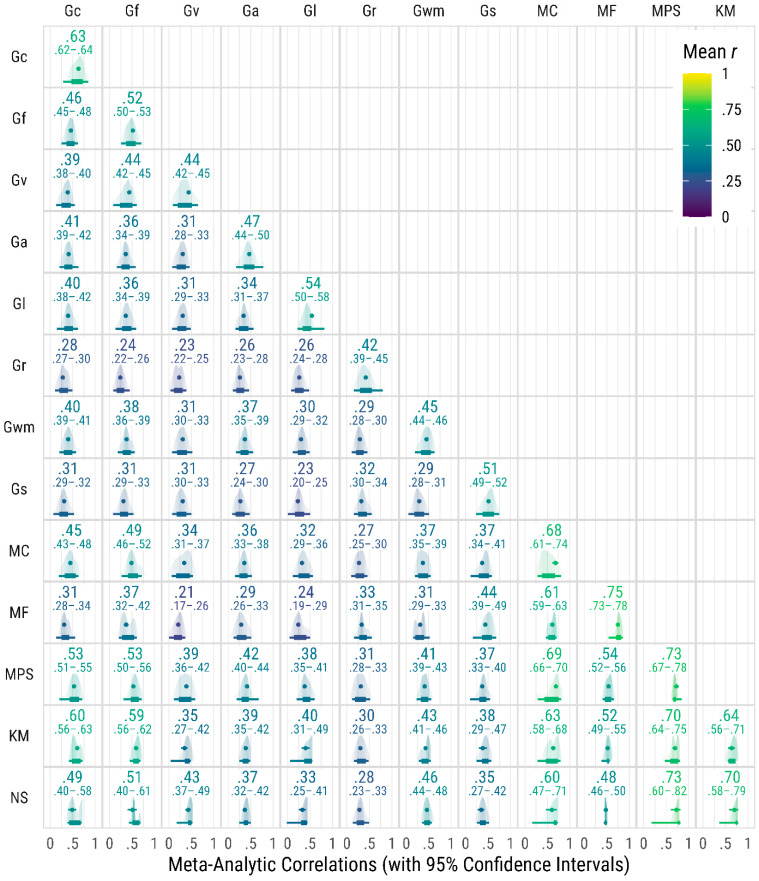
Meta-analytic correlations and standard errors between cognitive abilities and mathematics skills. Note. G*c* = Comprehension-Knowledge, G*f* = Fluid Reasoning, G*v* = Visual Spatial Processing, G*a* = Auditory Processing, G*l* = Long-Term Storage, G*r* = Retrieval Fluency, G*wm* = Short-Term Working Memory, G*s* = Processing Speed, MC = Math Calculation, MF = Math Fluency, MPS = Math Problem Solving, KM = Math Knowledge, NS = Number Sense.

The meta-analytic correlations among the cognitive abilities and mathematics skills are shown in Figure 1. All correlations were positive, statistically significant, and they were generally moderate (*r* = .30–.50) to large (*r* > .50) in size. Within the cognitive abilities, for each of the eight CHC abilities, the largest correlation was with tests of the same broad ability, providing some evidence that the tests are classified appropriately. Among the mathematics skills, most correlations are large in size (*r* > .50). The 95% confidence interval for the correlations is included in the table, showing the precision of each estimate.

### 3.3. Heterogeneity of Meta-Analytic Correlations

The *Q* values were statistically significant for all but two correlation pairs, including Math Fluency with Number Sense and Math Fluency with Math Knowledge, both of which only had five and 17 correlations, respectively. All other *Q* values were statistically significant, suggesting heterogeneity in the meta-analytic correlations. The *I*^2^ values were 85.5% on average, with a range of 2.2% to 96.3%. This suggests that most of the heterogeneity in correlations is due to true heterogeneity rather than chance.

**Figure 2:**
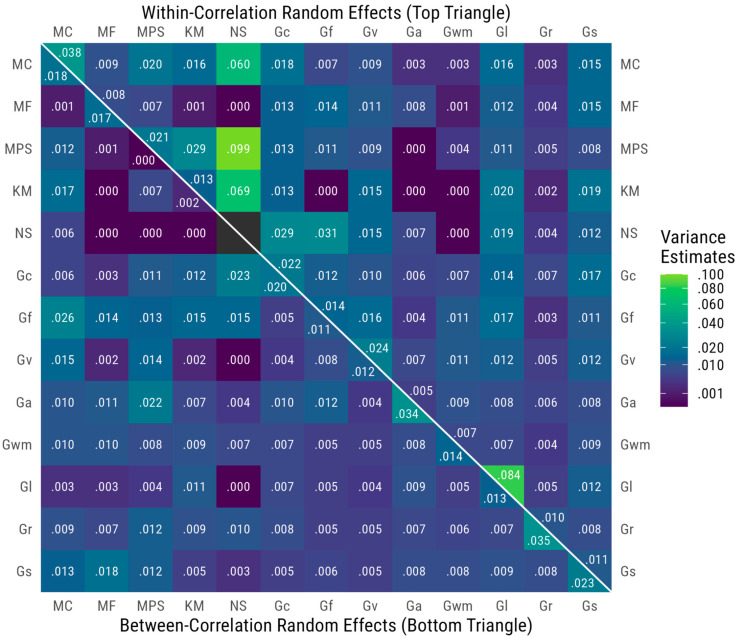
Level-2 and Level-3 random effects. Note. G*c* = Comprehension-Knowledge, G*f* = Fluid Reasoning, G*v* = Visual Spatial Processing, G*a* = Auditory Processing, G*l* = Long-Term Storage, G*r* = Retrieval Fluency, G*wm* = Short-Term Working Memory, G*s* = Processing Speed, MC = Math Calculation, MF = Math Fluency, MPS = Math Problem Solving, KM = Math Knowledge, NS = Number Sense.

The level-2 (between-sample random effect) and level-3 (within-sample random effect) variance estimates are included in Figure 2. The between variance was .01 on average, with a range of .00–.03. The within variance was .01 on average, with a range of .00–.10. This suggests that the correlation estimates across the different correlation matrices had some significant variability, as did the correlation estimates within correlation matrices.

### 3.4. SEM Using the Meta-Analytic Correlation Matrix

**Figure 3:**
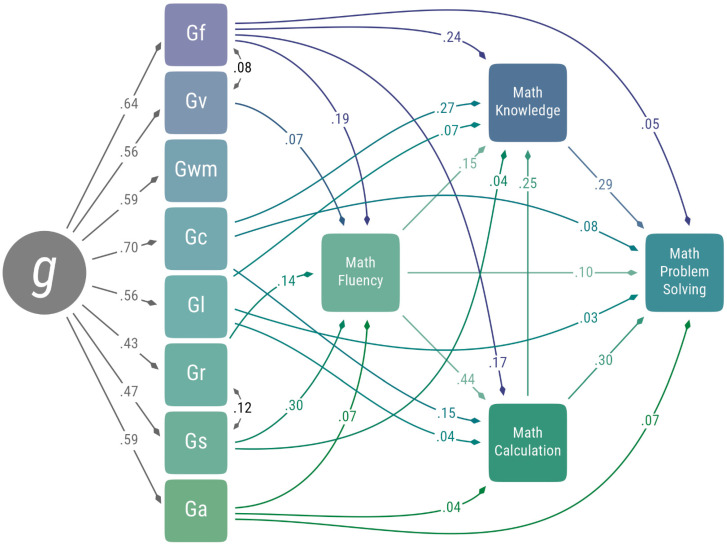
Meta-SEM for Cognitive–Mathematics Relations. Note*. g* = General Intelligence, G*c* = Comprehension-Knowledge, G*f* = Fluid Reasoning, G*v* = Visual Spatial Processing, G*a* = Auditory Processing, G*l* = Long-Term Storage, G*r* = Retrieval Fluency, G*wm* = Short-Term Working Memory, G*s* = Processing Speed.

Table 3 includes the results for the meta-SEM, and Figure 3 shows the model, only including paths with standardized coefficients greater than .05. Overall, the model had excellent fit, χ^2^ (18) = 5.76, *p* = .997, CFI = 1.00, TLI = 1.04, RMSEA = .000, SRMR = .011). Broad CHC ability loadings on *g* were moderate in size, ranging from .43 to .70. All path coefficients presented in the following paragraphs are standardized regression effects and can be interpreted as how much of a standard deviation increase in mathematics skills (the outcome) would be expected given a one standard deviation increase in a cognitive ability (the predictor). For instance, a standardized path coefficient of .35 between a cognitive ability and a mathematics skill would indicate that a one standard deviation increase in the cognitive ability would be associated with a .35 standard deviation increase in the mathematics skill.

When examining the paths from broad CHC abilities to mathematics skills, the direct effects were examined first. No direct effects from cognitive abilities to Number Sense were large in magnitude. There were moderate direct effects from cognitive abilities on Number Sense for Fluid Reasoning (β = .225), Short-Term Working Memory (β = .195), Visual Processing (β = .136), and Comprehension-Knowledge (β = .191). There were small direct effects from Processing Speed (β = .086) and Auditory Processing (β = .056), while all other effects were negligible in size.

There were large direct effects on Math Fluency from Number Sense (β = .302) and Processing Speed (β = .271), and moderate direct effects from Retrieval Fluency (β = .132) and Fluid Reasoning (β = .118). All other direct effects on Math Fluency were negligible. There were large direct effects on Math Calculation from Math Fluency (β = .369) and Number Sense (β = .269), with moderate direct effects from Fluid Reasoning (β = .124) and Comprehension-Knowledge (β = .105). All other cognitive abilities had negligible direct effects on Math Calculation.

The only large direct effect on Math Knowledge was from Number Sense (β = .336), with moderate direct effects from Comprehension-Knowledge (β = .226), Fluid Reasoning (β = .192), Math Calculation (β = .152), and Math Fluency (β = .108). There was one small direct effect from Long-Term Storage (β = .065), while all other direct effects were negligible. There was only one large direct effect for Math Problem Solving, which was from Number Sense (β = .350). There were moderate direct effects from Math Calculation (β = .240) and Math Knowledge (β = .151), and small direct effects from Math Fluency (β = .082), Comprehension-Knowledge (β = .072), and Auditory Processing (β = .063). All other direct effects on Math Problem Solving were negligible.

Next, the total effects of cognitive abilities on mathematics skills were examined (i.e., the sum of all direct and indirect effects). There were only direct effects on Number Sense, so the direct and total effects are the same. When considering Math Fluency, the large total effects included Number Sense (.302) and Processing Speed (.297), whereas moderate total effects were for Fluid Reasoning (.186) and Retrieval Fluency (.143), and small total effects were from Short-Term Working Memory (.077) and Auditory Processing (.072). All other total effects were negligible. The large total effects on Math Calculation were from Number Sense (.381), Math Fluency (.369), and Fluid Reasoning (.253), with moderate total effects from Comprehension-Knowledge (.175) and Processing Speed (.156), with small total effects from Short-Term Working Memory (.090), Auditory Processing (.077), and Long-Term Storage (.051). All other total effects were negligible.

The total effects that were large for Math Knowledge were Number Sense (.426), Fluid Reasoning (.326), and Comprehension-Knowledge (.322), with moderate total effects from Math Calculation (.152), Math Fluency (.164), Processing Speed (.124), and Short-Term Working Memory (.107), and a small total effect from Long-Term Storage (.083). Finally, the large total effects for Math Problem Solving were from Number Sense (.530) and Math Calculation (.263), with moderate total effects from Fluid Reasoning (.246), Comprehension-Knowledge (.234), Math Fluency (.195), Math Knowledge (.151), Auditory Processing (.113), and Processing Speed (.105), with small total effects from Short Term Working Memory (.096), Long-Term Storage (.074), Retrieval Fluency (.058), and Visual Processing (.055). All total effects were at least small in size for Math Problem Solving.

The effects of *g* on mathematics skills were indirect in this model. In other words, there was no direct effect of *g* on mathematics skills. Rather, *g* had direct effects on each of the broad cognitive abilities, which in turn had direct effects on each of the mathematics skills. Thus, the effects of *g* on mathematics skills are fully mediated by the broad cognitive abilities. These effects were all large, ranging from .418 for Math Fluency to .617 for Math Knowledge. The variance accounted for by cognitive abilities in more foundational mathematics skills was also large, ranging from 35.3% of the variance in Math Fluency to 42.9% of the variance in Number Sense. The variance accounted for in more advanced mathematics skills was large, ranging from 53.4% of the variance in Math Calculation to 67.4% of the variance in Math Problem Solving. Overall, cognitive abilities explained a substantial amount of variability in foundational mathematics skills (like Number Sense and Math Fluency) in this model, and cognitive abilities and foundational mathematics skills explained a substantial amount of variance in advanced mathematics skills (like Math Knowledge and Math Problem Solving).

Finally, a model was estimated without Number Sense because there were not very many Number Sense tests included in the meta-analytic correlation matrix (the number of correlation matrices with Number Sense ranged from 5 to 11), and they mostly came from the Woodcock–Johnson batteries. These results are in Table 4. When removing Number Sense from the model, this did not change the total effect of cognitive abilities on all other mathematics skills included in the model. While the direct effects were slightly larger, this is due to the removal of indirect effects from cognitive abilities through Number Sense on all other mathematics skills. Where there were some differences in total effects amongst mathematics skills, the primary differences were that the effects of foundational math skills were smaller than those of more advanced mathematics skills. Furthermore, examining the *R*^2^, it appears that removing Number Sense from the model reduced the variance accounted for in mathematics skills by approximately 5% across domains. Due to the small number of correlations used in the estimates of Number Sense, Figure 3 shows the model without Number Sense included, since these would represent the most robust findings of the meta-SEM.

### 3.5. Follow-Up Analysis with Narrow CHC Cognitive Abilities

The consistent relations between Comprehension-Knowledge and Fluid Reasoning with mathematics skills raised a post hoc question about whether narrow CHC cognitive abilities within some of the broad CHC abilities were differentially related to specific mathematics skills. The dataset included several narrow abilities that had adequate coverage to estimate meta-analytic correlations between narrow CHC abilities and mathematics skills. These included narrow abilities for Comprehension-Knowledge (Listening Ability, Communication Ability, Lexical Knowledge, and General Knowledge), Fluid Reasoning (Induction, General Sequential Reasoning [i.e., deductive reasoning], and Quantitative Reasoning), Visual Processing (Visualization and Visual Memory), and Short-Term Working Memory (Working Memory, Auditory Memory Span, and Visual Memory Span).

The correlation matrix for this follow-up analysis is in Table 5. All correlations were statistically significantly different from zero. On average, Mathematics Skills correlated most highly with Fluid Reasoning (average = .52, range = .31–.68), and then Comprehension-Knowledge (average = .43, range = .07–.62), Short-Term Working Memory (average = .40, range = .24–.52), and Visual Processing (average = .30, range = .12–.48). A meta-SEM was estimated with this correlation matrix. In this model, the narrow cognitive abilities are loaded on their respective broad factors, and then the broad factors are loaded on *g*. Mathematics skills were then regressed on all narrow cognitive abilities. In the initial model, the path from Quantitative Reasoning to all mathematics skills was large. A review of the tests that were included for Quantitative Reasoning indicated that many of them used numbers and required some knowledge of mathematics operations (e.g., Number Series tests on the WJ batteries). In order to reduce these potential method effects, the path from Quantitative Reasoning to all mathematics skills was removed. The model was reestimated, and the standardized paths from narrow cognitive abilities to mathematics skills are included in Table 6.

Several patterns emerged that were notable because they suggest differential relations between narrow cognitive abilities and specific mathematics skills. For Math Fluency, the largest effects were from Working Memory, General Sequential Reasoning, and General Knowledge. For Math Calculation, the largest effects were from General Knowledge, General Sequential Reasoning, and Visual Memory Span. For Math Knowledge, the largest effects were from Lexical Knowledge, Induction, Listening Ability, General Sequential Reasoning, Working Memory, and Auditory Memory Span. For Math Problem Solving, the largest effects were from General Knowledge, General Sequential Reasoning, Induction, and Listening Ability. The most consistently related narrow ability across mathematics skills was General Sequential Reasoning, followed by General Knowledge, Induction, and Working Memory. When examining indirect effects of the broad abilities, Comprehension-Knowledge had the largest consistent effects, followed by Fluid Reasoning and Short-Term Working Memory. Visual Processing was not highly related to any mathematics skills, and the indirect effects of *g* were large for all math skills, and were especially large for Math Knowledge and Math Problem Solving.

## 4. Discussion

This study combined over 550 unique correlation matrices and used over 47,000 correlations to examine the meta-analytic correlations between cognitive abilities and mathematics skills. The results showed that: (1) some cognitive abilities were more strongly and consistently related to mathematics skills than others, (2) the magnitude of the relationships between cognitive abilities and mathematics skills varied across different mathematics skills, and (3) a meta-SEM showed that Fluid Reasoning and Comprehension-Knowledge were the two largest and most consistent predictors of mathematics skills, with other cognitive abilities being small to moderate predictors of mathematics skills. Below, the results of this study are summarized, and how these results align with previous research and theoretical models of mathematics development, and the practical implications of these results are discussed.

### 4.1. Integrating Results with Previous Research and Theory

Fluid Reasoning was a moderate or large predictor of all mathematics skills in this study. These findings are consistent with previous research, including longitudinal studies (e.g., Green et al., 2017), cross-battery studies (Caemmerer et al., 2020), and other meta-analyses (e.g., Amland et al., 2025). Fluid Reasoning is thought to be related to mathematics skills because it serves as a foundational cognitive ability that is instrumental for problem solving, analyzing information, and solving unfamiliar situations in novel ways. In particular, the narrow ability Quantitative Reasoning, which involves solving mathematical problems without prior advanced knowledge of mathematics (Schneider & McGrew, 2018), is likely an important contributor to the relationship between Fluid Reasoning and mathematics skills. Importantly, the analysis with narrow CHC cognitive abilities showed that General Sequential Reasoning was consistently related to mathematics skills when Quantitative Reasoning was not included in the model. This suggests that the ability to use deductive reasoning skills is also important for mathematics skills, which is consistent with theoretical assertions that mathematics often requires the application of known rules and algorithms to novel problems. These results are consistent with LeFevre et al.’s (2010) research on the pathways model, where quantitative cognitive skills predict early numeracy and complex mathematical outcomes.

The effects of Comprehension-Knowledge on mathematics were consistently moderate-to-large except for Math Fluency, indicating that background knowledge and verbal skills are particularly strong predictors of math knowledge and problem solving. These findings are consistent with previous research that used co-normed test batteries like the WJ III (Floyd et al., 2003) and the WISC-V and the WIAT-III (Caemmerer et al., 2018). Comprehension-Knowledge is thought to be related to mathematical ability since general knowledge and communication skills are essential for learning and understanding mathematical concepts. Comprehension-Knowledge includes the depth and breadth of declarative and procedural knowledge, including language (Schneider & McGrew, 2018). These results are also consistent with LeFevre et al.’s (2010) research on the pathways model, where linguistic cognitive skills predict symbolic number system skills and complex mathematical outcomes. When examining the results with narrow CHC cognitive abilities, general knowledge was a relatively consistent predictor of mathematics skills, and lexical knowledge was an especially important predictor of Math Knowledge, highlighting the importance of vocabulary and language development for understanding mathematics concepts.

The effects of Short-Term Working Memory on mathematics skills were typically small except for Number Sense and Math Knowledge, where it was moderate. These findings are consistent with previous research that suggests Working Memory and mathematical ability are related (e.g., Amland et al., 2025; Peng et al., 2016). For instance, working memory has been linked to performance on national tests of mathematics skills scores (e.g., Jarvis & Gathercole, 2003). Working Memory is likely related to mathematical ability since mathematics requires manipulation of numbers when solving problems that cannot rely on Long-Term Storage or Comprehension-Knowledge (Coolen & Castronovo, 2023). The analysis with narrow CHC cognitive abilities indicated relations between Working Memory and Math Fluency and Math Knowledge, and Visual Memory Span with Math Calculation. These specific aspects of Short-Term Working Memory may be especially important when completing these tasks.

Processing Speed was generally a moderate predictor of mathematics skills except for Number Sense, where it was small. These findings are consistent with previous studies that used co-normed test batteries to determine the effects of general and broad abilities on mathematics skills, such as the WJ III normative sample (Taub et al., 2008). These results suggest that Processing Speed provides a foundational cognitive ability that underlies mathematics skills. It may be especially helpful to efficiently complete more automated tasks, such as determining the answers to simple math facts, rapid identification of small quantities (i.e., subitizing, Geary, 2011), or quickly identifying the appropriate procedures needed to solve a mathematics problem.

Consistent with previous research, several cognitive abilities had small to moderate relationships with mathematics. For instance, Retrieval Fluency was a moderate predictor of Math Fluency. Importantly, even though these are speeded tasks, Retrieval Fluency explained unique variance that was different from Processing Speed, which was a strong predictor of Math Fluency. Therefore, it is suggested that the ability to efficiently and accurately recall math facts from memory is a distinct skill that cannot be explained by Processing Speed alone. Additionally, Visual Processing, Auditory Processing, and Long-Term Storage had negligible to small effects on mathematics skills, with only Auditory Processing being a moderate predictor of Math Problem Solving and Visual Processing being a moderate predictor of Number Sense. These findings are consistent with previous research indicating that these cognitive abilities fall within the range of effects observed for mathematical ability in the present study (Amland et al., 2025; Calderón-Tena & Caterino, 2016; Villeneuve et al., 2019). Notably, when Number Sense was not in the model, Visual Processing still had negligible effects on other mathematics skills, suggesting Visual Processing may only be a predictor of Number Sense, but not other mathematical abilities. This was apparent when examining narrow CHC cognitive abilities as well, where narrow Visual Processing abilities were not related to any of the mathematics skills included in that analysis. Thus, Visual Processing may not act as a predictor of all mathematics skills, but only of Number Sense.

When examining the relations among mathematics skills, Number Sense was a strong predictor of all other mathematics skills. In this study, Number Sense was mapped onto the quantitative pathway of Dehaene’s pathways model based on the theoretical alignment of the construct of Number Sense with this pathway. While the current study did not necessarily test whether this alignment was accurate, these findings are consistent with previous longitudinal studies that suggest that Number Sense is a strong predictor of mathematics as children age (Starr et al., 2013). However, caution should be taken when interpreting this finding because there was a small number of tests used to estimate the correlations for Number Sense and other cognitive abilities and Mathematics Skills. Given the relatively few Number Sense tests included in the meta-analytic correlation matrix, which mostly came from the Woodcock–Johnson test batteries, it is possible that the predictive weight of Number Sense on mathematics skills would differ when using a greater number and variety of test batteries. This would be an important area for future research, and the development of other Number Sense tests would be helpful in clarifying whether this relation would hold with a greater variety of tests and across different samples. Despite this limitation, this finding highlights the potential importance of evaluating mathematics skills using a hierarchical lens, where Number Sense and Math Fluency serve as strong scaffolding for more advanced mathematics skills, including Math Calculation, Math Knowledge, and Math Problem Solving. This hierarchical lens is supported by other meta-analytic work (Y. Liu et al., 2025) and supports the need to examine and understand mathematics skills through a hierarchical model (Xu et al., 2023) rather than conceptualizing mathematics as correlated academic skills that do not have direct, causal effects on each other.

Finally, this study has broader implications for theories of intelligence. Previous work using an integrated model for cognitive-reading relations highlighted the importance of the interconnectedness of cognitive abilities and reading, showing that different narrow reading skills are predicted by different cognitive abilities, and foundational reading skills are predictors of complex reading skills (Hajovsky et al., 2025). These same patterns were found in the current study. Specifically, these results demonstrate how general, broad, and narrow cognitive abilities within the CHC framework collectively predict mathematical skills as an integrated system. As such, theories of intelligence should continue to reflect these complex patterns of cascading interconnectedness. Most previous research on cognitive–mathematics relations has focused on the relations between broad cognitive abilities and broad mathematics skills (e.g., Caemmerer et al., 2023; Taub et al., 2008). The current study provides a closer examination of relations between both broad and narrow cognitive abilities and narrow mathematics skills, providing some evidence that narrow cognitive abilities have differential relations with narrow mathematics skills, emphasizing the potential importance of considering specific cognitive abilities and processes when understanding mathematics skill development.

These results may also be interpreted through other theoretical frameworks of intelligence, such as mutualism (Peng & Kievit, 2020), where cognitive abilities and academic achievement mutually influence each other in a bidirectional manner across development. A strong test of this framework would require longitudinal data where reciprocal relations could be modeled, but the current results suggest that foundational skills are predictive of more complex skills and may provide preliminary evidence for models that test bidirectional relations more specifically.

In addition, these results could be evaluated within the framework of process overlap theory (Kovacs & Conway, 2016). While process overlap theory has focused primarily on explaining why positive manifold exists among cognitive ability tests, it suggests that one reason why scores on cognitive ability and mathematics tests are positively correlated is that accurate test performance relies on similar cognitive and executive processes. For example, the finding that Fluid Reasoning (especially the narrow cognitive ability of General Sequential Reasoning) was consistently related to mathematics skills suggests that mathematics relies on cognitive processes that require this type of reasoning. Importantly, this is different from findings for reading, where Auditory Processing is consistently related to different reading skills, but Fluid Reasoning is not consistently related to different reading skills (e.g., Hajovsky et al., 2025). When considering process overlap theory, these differential relations may help clarify that different academic skills are related to different cognitive processes due to the overlap of different cognitive processing requirements needed to engage in those academic skills. Overall, our results provide some helpful insights into how and why some cognitive abilities are predictive of mathematics skills and may help researchers continue to develop theories of intelligence that explain the relationships between cognitive abilities and academic skills.

### 4.2. Practical Implications for School-Based Evaluations

These results have clear implications for how practitioners evaluate mathematical learning and design assessment batteries and argue for moving beyond a single mathematics achievement score toward targeted cognitive measures that may help identify whether mathematics difficulties are due to conceptual, procedural, or efficiency-based reasons. Ultimately, this may help guide instructional decisions that are more precisely related to the reasons why a person has difficulty learning mathematics skills.

The findings indicate that Fluid Reasoning is a strong predictor of a range of mathematics skills. Brief measures of Fluid Reasoning can help clarify whether a student’s mathematics difficulties stem from limitations in reasoning rather than from instructional gaps. Specifically, the narrow ability of General Sequential Reasoning seems to be especially important to evaluate when mathematics concerns arise. The focus on deductive reasoning skills and the application of logic and rule-based algorithmic problem solving that is included in these types of tests (e.g., Figure Weights on the Wechsler scales) can help clarify if someone is able to apply these skills in novel situations, and if someone is having difficulty with General Sequential Reasoning tasks, it may show that those difficulties may contribute to challenges learning mathematics. Consequently, students who present difficulty with Fluid Reasoning may face an increased risk of also having challenges across all areas of mathematical ability. Using this hierarchical model as a framework for comprehensive evaluations for mathematical difficulty allows practitioners to identify underlying cognitive concerns related to general problem-solving ability.

Comprehension-Knowledge was also a strong predictor of a range of mathematics skills, particularly Math Knowledge and Math Problem-Solving. Language and conceptual knowledge should be assessed alongside mathematics skills. Since Comprehension-Knowledge reflects a student’s depth of acquired knowledge and verbal reasoning, these results suggest that assessing this cognitive ability is essential for a student’s overall mathematical profile. For instance, the role of Comprehension-Knowledge in predicting Math Knowledge and Math Problem Solving may inform practitioners of areas of weakness in mathematical concepts and, consequently, their application. Thus, individuals who present difficulty with Comprehension-Knowledge may also struggle to navigate conceptual demands that are instrumental for Math Problem Solving. Using this hierarchical framework, practitioners can directly inform academic interventions that specifically target conceptual knowledge of mathematics, such as breaking down complex terms through scaffolding.

Beyond Fluid Reasoning and Comprehension-Knowledge, it is also important to assess Processing Speed and Short-Term Working Memory. Although the magnitude of the relations between these two cognitive abilities was not as consistently strong as Fluid Reasoning and Comprehension-Knowledge, aspects of these abilities suggested they may play an important role as foundational components of mathematical ability. Processing Speed and Short-Term Working Memory reflect the efficiency and manipulation of cognitive operations, which suggest that assessing these domains is essential for characterizing a student’s overall mathematical profile. Notably, Processing Speed had a small effect on Number Sense, and Short-Term Working Memory had a moderate effect on Number Sense, indicating that foundational numerical concepts may rely more on active manipulation than on cognitive speed. Therefore, students who present difficulty in these efficiency-based procedures may also struggle with tasks that require mental math (e.g., quickly solving for 3 + 4) or multi-step procedures (e.g., dividing a three-digit number by a two-digit number). Through the present hierarchical framework, practitioners may identify these cognitive concerns, which directly inform appropriate academic accommodations, such as extended time for procedural tasks, or interventions such as Incremental Rehearsal, where students learn math facts through a systematic drill and practice that intersperses known and unknown math facts to build mathematical fluency (Burns, 2005).

While Visual Processing generally presented little predictive value for broad mathematical abilities, it emerged as a moderate predictor for Number Sense. These results suggest that Visual Processing may be important for the early acquisition of mathematics skills that are not captured in more advanced tasks. If a student presents difficulty in early numerical development, practitioners should prioritize assessing for Visual Processing to better inform on underlying spatial or imagery-related barriers. This can also inform interventions that utilize visual–spatial supports to foster number concept development, such as the Concrete–Representational–Abstract math approach, where a number concept is visualized through concrete materials such as blocks and progresses to conceptualize the magnitudes of such numbers through abstract thinking (Ebner et al., 2025).

Overall, the assessment of mathematics concerns should routinely include brief, validated measures of Fluid Reasoning, Comprehension-Knowledge, Processing Speed, and Short-Term Working Memory. These broad abilities explained substantial variance in both foundational outcomes (i.e., Number Sense, Math Fluency) and advanced outcomes (i.e., Math Calculation, Math Knowledge, Math Problem Solving). In practice, Comprehension-Knowledge is most informative for identifying conceptual gaps, such as misunderstandings of mathematics vocabulary or concepts, and these results show that Comprehension-Knowledge strongly predicts Math Knowledge and Math Problem-Solving. Processing Speed may be most informative for identifying inefficient processing (e.g., slow or error-prone fluency) given the large direct and total effects of Processing Speed on Math Fluency. Fluid Reasoning and Short-Term Working Memory may be most informative for identifying limitations in problem solving (e.g., difficulty with novel, multi-step, or relational problems). Specifically, Fluid Reasoning had moderate-to-large effects across mathematics outcomes, and Short-Term Working Memory was predictive of Number Sense, with downstream effects on Math Problem Solving.

Understanding how mathematics skills are related to each other is also essential for the evaluation of mathematics difficulties, and this framework is presented through the integrated model. It is essential for practitioners to include curriculum-based mathematics probes to distinguish among conceptual misunderstandings, processing inefficiencies, and problem-solving limitations. Interpretation and error analysis should be structured around the hierarchical math model used here. Linking error analysis to cognitive test results may then help determine whether errors stem from conceptual misunderstanding (linked to Comprehension-Knowledge), reasoning deficits (linked to Fluid Reasoning), or procedural/efficiency problems (linked to Processing Speed and/or Short-Term Working Memory). For instance, practitioners should initially examine Number Sense and Math Fluency as potential bottlenecks that hinder the development of more complex mathematics skills. These two mathematics skills may serve as important variables for developing complex mathematics skills, though conclusions about this should be tempered, given the smaller number of correlations of Number Sense with the cognitive abilities and other mathematics skills. The early screening of Number Sense and reasoning skills may be especially helpful for identifying early mathematics difficulties. This essential mathematics skill that develops and predicts complex mathematics skills may be akin to phonological processing for reading, so further research and focus on Number Sense may be helpful for preventing future mathematics difficulties.

Consequently, evaluating Math Calculation, Math Knowledge, and Math Problem Solving should be informed by understanding the mathematics skills that underlie them, namely Number Sense and Math Fluency. This approach allows practitioners to better understand the difficulties in more advanced mathematics skills. For example, if a student is having difficulty with Math Problem Solving, is it because they have difficulties understanding the logic needed to set up the problem, or because of calculation difficulties? If they can set up the problem correctly, but calculate the answer incorrectly, then the issue may not be due to understanding math problem solving, but in performing calculations correctly. Similarly, if they do not set up the problem correctly but are able to calculate the answer correctly from the incorrect problem, that could indicate their calculation skills are adequate, and they need help with the problem-solving aspects of mathematics. Ultimately, if math knowledge appears to be what is most difficult for the student, practitioners can target interventions for problem representation and strategic categorization; similarly, if calculation appears to be the core issue, interventions should focus on procedural fluency and the use of developmentally appropriate strategies. These difficulties are not apparent from test scores alone, and careful error analysis may help determine where the difficulty lies, whether it is an issue with procedural execution, linguistic barriers, or calculation. By recognizing how these skills build upon one another, practitioners can better identify the primary point of difficulty of a student’s mathematical profile and ultimately support the transition from basic numerical fluency to more sophisticated problem-solving strategies.

From there, recommendations may be tailored accordingly (e.g., conceptual scaffolding vs. fluency drills vs. extended time). This approach may reduce misattribution of reasons for mathematics difficulties, such as stating that a student has a mathematics learning disability that manifests as calculation difficulties when the primary issue is language comprehension that has affected their ability and leads to more targeted interventions. The selective evaluation can subsequently inform the design of academic interventions that target the application of existing rules in novel ways to solve new problems, which, in consequence, support all mathematical abilities too (e.g., *Solve it*! Intervention, Montague et al., 2000).

### 4.3. Limitations and Future Directions

There are several important limitations to this study that are worth considering and can help guide future work in this area. While meta-analyses mitigate the sampling error inherent in individual studies, these data are derived from normative samples that do not include clinical populations such as students with specific learning disabilities in mathematics. Future research should apply this model to clinical subgroups to determine if the predictive power of broad CHC cognitive abilities for mathematics skills remains the same across clinical and non-clinical populations. Second, this study only includes English-speaking people from the United States, so it is unknown if these results would generalize to cognitive and mathematics skills across other languages and cultures. Third, although the meta-analytic correlation estimates in this study were robust and combined together a substantial number of correlations (well into the thousands in some cases), there was substantial heterogeneity in those correlations, suggesting that different test batteries, subtests, and samples produce a distribution of correlations, and there is not necessarily one “true” correlation amongst the constructs examined in this study. A full moderator analysis was beyond the scope of this study, though the heterogeneity could be due to a number of factors, including developmental age, different test battery families, and how the tests included on them measure various cognitive abilities and mathematics skills, or the year that the test was published. A review of the *I*^2^ values did not suggest any substantive patterns in correlations that had high and low *I*^2^ values, although one pattern that appeared was that within-ability correlations tended to have higher *I*^2^ values (i.e., the within-ability correlations for seven of the twelve variables had *I*^2^ values > .95). Since this analysis specifically excluded correlations among subtests that were essentially the same (e.g., correlations between Matrix Reasoning across two different versions of the Wechsler scales), this may highlight the range of different ways in which cognitive abilities and mathematics skills are measured across different test batteries.

Related to the moderator analysis, the examination of developmental differences in these relations was not tested. The results for this analysis are essentially average effects between cognitive abilities and mathematics skills across the lifespan, although previous research has found developmental differences in these relationships (e.g., Niileksela et al., 2025; Villeneuve et al., 2019). It is likely that some of the relationships found may be larger or smaller at different developmental levels, and they will likely change with development and exposure to mathematics instruction.

One assumption of the integrated mathematics model is that the effects of broad CHC cognitive abilities on advanced mathematics skills operate, at least in part, via basic mathematics skills (i.e., a mediation pathway). Because our meta-analytic correlations are cross-sectional and aggregated from multiple independent test manuals, these data cannot establish temporal ordering or causal mediation. Cross-sectional indirect-effect estimates are vulnerable to bias from unmeasured confounding variables and misspecified temporal sequencing (Maxwell & Cole, 2007; Pearl, 2000). For example, socioeconomic status, prior instructional exposure, or language proficiency could influence both the mediator measures (e.g., Number Sense) and advanced math outcomes, which may inflate indirect effects. To avoid overstating causal claims, the current mediation results may be consistent with mediation hypotheses rather than as proof of sequential causal processes. In other words, in this study, the indirect effects of broad CHC abilities on advanced mathematics skills are assumed to occur simultaneously rather than sequentially, and when looking at mediation, it is best to have longitudinal data.

We used the pathways model (Dehaene et al., 2005) to theoretically map Number Sense and Math Fluency onto the quantitative and linguistic pathways, respectively. While our meta-analytic findings are consistent with these mappings, we could not directly test them. Additionally, this study used CHC theory as a theoretical anchor, but there are other cognitive ability taxonomies and models, such as the Planning, Attention, Simultaneous, and Sequential (PASS) theory that could also be used, where previous research suggests some of these cognitive processes are related to mathematics skills (e.g., Georgiou et al., 2020) and can help inform mathematics intervention (Naglieri & Johnson, 2000), although PASS cognitive processes have also been linked to CHC theory (Kranzler & Keith, 1999; Kranzler et al., 2000). Related to the focus on CHC theory as a theoretical anchor, the classification of tests into CHC theory primarily relied on a combination of expert opinion and previous research that is available, so not all classifications are wholly empirically derived. For instance, some tests have a substantial amount of research (e.g., Wechsler scales) that help inform test classifications, but others have less research (e.g., Feifer Assessment of Mathematics), and more reliance must be put on the tasks and task demands to help determine CHC classifications.

### 4.4. Conclusions

This study consolidates a very large number of datasets collected from the standardization and validity samples from norm-referenced standardized test batteries to clarify how cognitive abilities predict mathematics skills and how basic mathematics skills predict advanced mathematics skills. Meta-analysis is a helpful method for summarizing the substantial amount of data available for cognitive–mathematics relations. Several results are consistent with previous research, and this work helps bring together this large body of work into a general picture of how cognitive abilities predict mathematics skills. Ideally, this research can be used to clarify how cognitive abilities and mathematics skills are related to each other in a way that helps continue the development of theoretical models of mathematics and the development of instructional strategies that may be used to improve mathematics achievement for struggling students.

## Figures and Tables

**Table 1:** Number of correlations used to estimate meta-analytic correlations.

	Gc	Gf	Gv	Ga	Gl	Gr	Gwm	Gs	MC	MF	MPS	KM	NS
G*c*	3197												
G*f*	1831	400											
G*v*	3170	1219	1125										
G*a*	852	386	400	190									
G*l*	1257	679	1791	384	495								
G*r*	1057	519	541	381	496	331							
G*wm*	3778	1582	2756	566	1350	817	1619						
G*s*	2215	824	1427	342	556	503	1929	501					
MC	472	138	168	130	132	166	204	122	111				
MF	391	98	94	104	91	177	157	108	118	52			
MPS	442	132	152	139	128	162	205	122	185	109	13		
KM	130	28	34	25	23	53	48	25	133	17	52	26	
NS	68	26	25	28	29	54	47	27	16	5	12	12	0

Note. G*c* = Comprehension-Knowledge, G*f* = Fluid Reasoning, G*v* = Visual Spatial Processing, G*a* = Auditory Processing, G*l* = Long-Term Storage, G*r* = Retrieval Fluency, G*wm* = Short-Term Working Memory, G*s* = Processing Speed, MC = Math Calculation, MF = Math Fluency, MPS = Math Problem Solving, KM = Math Knowledge, NS = Number Sense.

**Table 2:** The number of correlation matrices that included correlations used to estimate meta-analytic correlations.

	G*c*	G*f*	G*v*	G*a*	G*l*	G*r*	G*wm*	G*s*	MC	MF	MPS	KM	NS
G*c*	384												
G*f*	216	150											
G*v*	273	222	263										
G*a*	82	49	55	34									
G*l*	123	111	160	48	126								
G*r*	112	89	107	65	70	76							
G*wm*	252	199	278	63	142	106	220						
G*c*	179	154	198	44	70	85	182	133					
MC	135	53	56	57	43	57	59	46	14				
MF	59	21	21	39	19	47	25	21	55	20			
MPS	110	51	55	62	46	59	57	47	95	55	10		
KM	28	7	9	9	9	10	10	7	13	6	12	4	
NS	10	7	10	10	10	11	10	7	7	5	7	7	0

Note. G*c* = Comprehension-Knowledge, G*f* = Fluid Reasoning, G*v* = Visual Spatial Processing, G*a* = Auditory Processing, G*l* = Long-Term Storage, G*r* = Retrieval Fluency, G*wm* = Short-Term Working Memory, G*s* = Processing Speed, MC = Math Calculation, MF = Math Fluency, MPS = Math Problem Solving, KM = Math Knowledge, NS = Number Sense.

**Table 3:** Standardized effects for the integrated cognitive–mathematics model using a meta-analytic correlation matrix.

	Number Sense Direct Effect (Indirect, Total Effect)	Math Fluency Direct Effect (Indirect, Total Effect)	Math Calculation Direct Effect (Indirect, Total Effect)	Math Knowledge Direct Effect (Indirect, Total Effect)	Math Problem Solving Direct Effect (Indirect, Total Effect)
Comprehension-Knowledge	**.191** **(--, --)**	*−.006* *(.058, .052)*	**.105** **(.070, .175)**	**.226** **(.096, .322)**	**.072** **(.162, .234)**
Fluid Reasoning	**.225** **(--, --)**	**.118** **(.068, .186)**	**.124** **(.129, .253)**	**.192** **(.134, .326)**	**.042** **(.204, .246)**
Visual Processing	**.136** **(--, --)**	*−.109* *(.041, −.068)*	*.022* *(.011, .033)*	*−.084* *(.043, −.041)*	*.011* *(.044, .055)*
Auditory Processing	*.056* *(--, --)*	*.055* *(.017, .072)*	*.035* *(.042, .077)*	*.002* *(.038, .040)*	**.063** **(.050, .113)**
Long-Term Storage	*.025* *(--, --)*	*.013* *(.008, .021)*	*.037* *(.014, .051)*	*.065* *(.018, .083)*	*.039* *(.035, .074)*
Retrieval Fluency	*.036* *(--, --)*	**.132** **(.011, .143)**	*−.016* *(.062, .047)*	*−.002* *(.035, .033)*	*.018* *(.040, .058)*
Short-Term Working Memory	**.195** **(--, --)**	*.018* *(.059, .077)*	*.009* *(.081, .090)*	**.019** **(.087, .107)**	*−.016* *(.112, .096)*
Processing Speed	*.086* *(--, --)*	**.271** **(.026, .297)**	**.023** **(.133, .156)**	**.039** **(.085, .124)**	*−* **.005** **(.110, .105)**
Number Sense	--	**.302** **(--, --)**	**.269** **(.112, .381)**	**.336** **(.090, .426)**	**.350** **(.180, .530)**
Math Fluency	--	--	**.369** **(--, --)**	**.108** **(.056, .164)**	**.082** **(.113, .195)**
Math Calculation	--	--	--	**.152** **(--, --)**	**.240** **(.023, .263)**
Math Knowledge	--	--	--	--	**.151** **(--, --)**
Indirect Effect of *g*	.571	.418	.523	.617	.591
*R* ^2^	.426	.353	.534	.655	.674

Note. Cells with no shading and italicized text are negligible effects, cells with light gray shading and italicized text are small effects, cells with moderate gray shading and bold text are moderate effects, and cells with dark gray shading and bold text are large effects.

**Table 4:** Standardized effects for the integrated cognitive–mathematics model using a meta-analytic correlation matrix without Number Sense.

	Math Fluency Direct Effect (Indirect, Total Effect)	Math Calculation Direct Effect (Indirect, Total Effect)	Math Knowledge Direct Effect (Indirect, Total Effect)	Math Problem Solving Direct Effect (Indirect, Total Effect)
Comprehension-Knowledge	*.052* *(--, --)*	**.153** **(.022, .175)**	**.271** **(.051, .322)**	**.080** **(.154, .234)**
Fluid Reasoning	**.186** **(--, --)**	**.172** **(.081, .253)**	**.236** **(.090, .326)**	**.054** **(.192, .246)**
Visual Processing	*−.068* *(--, --)*	*.063* *(−.030, .033)*	*−.039* *(−.002, −.041)*	.064 (−.009, .055)
Auditory Processing	*.072* *(--, --)*	*.045* *(.032, .077)*	*.011* *(.030, .040)*	**.071** **(.043, .114)**
Long-Term Storage	*.021* *(--, --)*	*.042* *(.009, .051)*	*.068* *(.016, .083)*	*.032* *(.042, .074)*
Retrieval Fluency	**.143** **(--, --)**	*−.015* *(.062, .047)*	*.000* *(.033, .033)*	*.020* *(.039, .058)*
Short-Term Working Memory	*.077* *(--, --)*	*.056* *(.034, .090)*	**.073** **(.034, .107)**	*.030* *(.067, .096)*
Processing Speed	**.297** **(--, --)**	**.026** **(.130, .156)**	**.041** **(.083, .124)**	***−*.009** **(.114, .105)**
Math Fluency	--	**.436** **(--, --)**	**.150** **(.107, .257)**	**.104** **(.208, .312)**
Math Calculation	--	--	**.247** **(--, --)**	**.303** **(.073, .376)**
Math Knowledge	--	--	--	**.295** **(--, --)**
Indirect Effect of *g*	.418	.523	.617	.591
*R* ^2^	.301	.496	.600	.622

Note. Cells with no shading and italicized text are negligible effects, cells with light gray shading and italicized text are small effects, cells with moderate gray shading and bold text are moderate effects, and cells with dark gray shading and bold text are large effects.

**Table 5:** Meta-analytic correlations between select CHC narrow abilities and mathematics skills.

	Comprehension-Knowledge Narrow Abilities				Fluid Reasoning Narrow Abilities			Short-Term Working Memory Narrow Abilities			Visual Processing Narrow Abilities		Mathematics Skills				
	LS	CM	VL	K0	I	RG	RQ	Wa	Wc	Wv	Vz	MV	MC	MF	MPS	KM	NS
LS	1.00																
CM	.56	1.00															
VL	.59	.63	1.00														
K0	.51	.31	.70	1.00													
I	.44	.32	.47	.46	1.00												
RG	.44	.34	.46	.47	.51	1.00											
RQ	.42	.41	.50	.45	.52	.48	1.00										
Wa	.42	.45	.39	.39	.33	.31	.31	1.00									
Wc	.39	.35	.41	.41	.41	.41	.47	.43	1.00								
Wv	.27	.23	.36	.32	.39	.32	.46	.33	.40	1.00							
Vz	.35	.25	.43	.44	.48	.46	.47	.28	.37	.35	1.00						
MV	.31	.22	.32	.28	.36	.32	.30	.22	.28	.37	.33	1.00					
MC	.40	.34	.45	.52	.43	.47	.63	.32	.42	.40	.39	.26	1.00				
MF	.29	.23	.31	.35	.31	.34	.52	.24	.40	.26	.24	.19	.61	1.00			
MPS	.50	.41	.56	.57	.51	.52	.63	.38	.45	.36	.45	.28	.69	.54	1.00		
KM	.51	.36	.62	.56	.58	.51	.66	.41	.49	.43	.39	.23	.63	.52	.70	1.00	
NS	.41	.07	.55	.57	.47	.52	.68	.44	.52	.44	.48	.12	.60	.48	.73	.70	1.00

Note. LS = Listening Ability, CM = Communication Ability, VL = Lexical Knowledge, K0 = General Knowledge, I = Induction, RG = General Sequential Reasoning, RQ = Quantitative Reasoning, Wa = Auditory Memory Span, Wc = Working Memory, Wv = Visual Memory Span, Vz = Visualization, MV = Visual Memory, MC = Math Calculation, MF = Math Fluency, MPS = Math Problem Solving, KM = Math Knowledge, NS = Number Sense.

**Table 6:** Standardized effects from Meta-SEM between narrow cognitive abilities and mathematics skills.

	Math Fluency β	Math Calculation β	Math Knowledge β	Math Problem Solving β
*Narrow Abilities*				
*Comprehension-Knowledge Narrow*				
Listening Ability	*.032*	*.022*	**.139**	**.101**
Communication Ability	*.012*	*.085*	*−.170*	*.059*
Lexical Knowledge	*−.038*	*−.065*	**.341**	*.064*
General Knowledge	**.174**	**.299**	*.057*	**.223**
*Fluid Reasoning Narrow*				
Induction	*.066*	*.073*	**.249**	**.129**
General Sequential Reasoning	**.135**	**.191**	**.136**	**.167**
*Short-Term Working Memory Narrow*				
Auditory Memory Span	*.049*	*.039*	**.122**	*.065*
Working Memory	**.221**	*.083*	**.129**	*.079*
Visual Memory Span	*−.010*	**.112**	*.070*	*.004*
*Visual Processing Narrow*				
Visualization	*−.037*	*.052*	*−.045*	*.086*
Visual Memory	*.031*	*.009*	*−.092*	*.003*
*Indirect Effects of Broad Abilities and g*				
Comprehension-Knowledge	**.136**	**.258**	**.316**	**.347**
Fluid Reasoning	**.140**	**.183**	**.271**	**.207**
Short-Term Working Memory	**.177**	**.147**	**.203**	*.096*
Visual Processing	*−.009*	*.039*	*−.074*	*.059*
*g*	**.393**	**.553**	**.627**	**.620**

Note. Cells with no shading and italicized text are negligible effects, cells with light gray shading and italicized text are small effects, cells with moderate gray shading and bold text are moderate effects, and cells with dark gray shading and bold text are large effects.

## Data Availability

The data are not publicly available but are located within publisher test manuals available to those who have permission to administer such tests.

## Supplementary Materials

The following supporting information can be viewed at the links provided. Test batteries used for meta-analysis: https://drive.google.com/file/d/1Ju5ImvlRal7ChO37ujgsrckpw5pOgcvP/view?usp=drive_link (accessed on 20 June 2026). All correlations, sample sizes, subtests, and CHC broad and narrow categorizations: https://drive.google.com/file/d/1C0L0Pz4vJlMhgqRJzDaeubrx2KT5RtqE/view?usp=sharing (accessed on 20 June 2026).
